# Abnormal global functional network connectivity and its relationship to medial temporal atrophy in patients with amnestic mild cognitive impairment

**DOI:** 10.1371/journal.pone.0179823

**Published:** 2017-06-26

**Authors:** Can Sheng, Mingrui Xia, Haikuo Yu, Yue Huang, Yan Lu, Fang Liu, Yong He, Ying Han

**Affiliations:** 1 Department of Neurology, XuanWu Hospital of Capital Medical University, Beijing, P. R. China; 2 Department of Neurology, the First Hospital of Tsinghua University, Beijing, P. R. China; 3 Center of Alzheimer’s Disease, Beijing Institute for Brain Disorders, Beijing, P. R. China; 4 State Key Laboratory of Cognitive Neuroscience and Learning, Beijing Normal University, Beijing, P. R. China; 5 Beijing Key Laboratory of Brain Imaging and Connectomics, Beijing Normal University, Beijing, P. R. China; 6 IDG/McGovern Institute for Brain Research, Beijing Normal University, Beijing, P. R. China; 7 Department of Rehabilitation, XuanWu Hospital of Capital Medical University, Beijing, P. R. China; 8 School of Medical Sciences, Faculty of Medicine, UNSW Australia, New South Wales, Australia; 9 Department of Ophthalmology, XuanWu Hospital of Capital Medical University, Beijing, P. R. China; 10 Beijing Institute of Geriatrics, Beijing, P. R. China; 11 National Clinical Research Center for Geriatric Disorders, Beijing, P. R. China; 12 PKU Care Rehabilitation Hospital, Beijing, P. R. China; University of Manchester, UNITED KINGDOM

## Abstract

**Background:**

Amnestic mild cognitive impairment (aMCI), which is recently considered as a high risk status for developing Alzheimer’s disease (AD), manifests with gray matter atrophy and increased focal functional activity in the medial temporal lobe (MTL). However, the abnormalities of whole-brain functional network connectivity in aMCI and its relationship to medial temporal atrophy (MTA) remain unknown.

**Methods:**

In this study, thirty-six aMCI patients and thirty-five healthy controls (HCs) were recruited. Neuropsychological assessments and MTA visual rating scaling were carried out on all participants. Furthermore, whole brain functional network was constructed at voxel level, and functional connectivity strength (FCS) was computed as the sum of the connections for each node to capture its global integrity. General linear model was used to analyze the FCS values differences between aMCI and HCs. Then, the regions showing significant FCS differences were adopted as the imaging markers for discriminative analysis. Finally, the relationship between FCS values and clinical cognitive scores was correlated in patients with aMCI.

**Results:**

Comparing to HCs, aMCI exhibited significant atrophy in the MTL, while higher FCS values within the bilateral MTL regions and orbitofrontal cortices. Notably, the right hippocampus had the highest classification power, with the area under receiver operating characteristics (ROC) curve (AUC) of 0.790 (confidence interval: 0.678, 0.901). Moreover, FCS values of the right hippocampus and the left temporal pole were positively correlated with the cognitive performance in aMCI.

**Conclusion:**

This study demonstrated significantly structural atrophy and raised global functional integrity in the MTL, suggesting simultaneous disruption and compensation in prodromal AD. Increased intrinsic functional connectivity in the MTL may have the potential to discriminate subjects with tendency to develop AD.

## Introduction

Alzheimer’s disease (AD), characterized by progressive decline of memory and other cognitive domains, is the most common form of neurodegenerative disorders leading to dementia [1, 2]. Amnestic mild cognitive impairment (aMCI), which also has significant episodic memory loss, is widely considered as a high risk factor for developing AD. Most aMCI patients will progress to AD at a rate of 10% to 15% per year [3] and aMCI patients without comorbidities has 8.5 times higher risk of converting to dementia than possible non-aMCI [4]. Given lack of effective treatment at the stage of dementia recently, investigation on brain abnormalities to delineate AD-related disease progression and achieve early diagnosis at the stage of aMCI are of great values.

The medial temporal lobe (MTL), which is one of the main components of default mode network (DMN), plays a critical role in the processing of episodic memory [5]. MTL is considered to be initially targeted by AD-related pathology [6] and presents significant gray matter atrophy both in AD and aMCI patients [7–10]. However, numerous studies have shown that local functional activity within the MTL regions is increased during memory-related processes [11–14]. Sperling et al. found that MTL activity is paradoxically increased in patients with MCI compared to controls [15], which may compensate for the evolving disruption of the MTL in order to achieve the performance of control subjects [11]. Dickerson et al. have confirmed the relationship between greater extent of activation within the hippocampal and parahippocampal gyrus and better memory performance in MCI patients [13]. Additionally, using resting-state functional MRI (rs-fMRI), similar changes of MTL intrinsic activity were reported in current studies. In AD dementia, hippocampus exhibited raised local functional connectivity [16] and intrinsic activity in several right MTL subregions was increased, latter of which was also at-trend associated with impaired delayed recall [17]. Comparable abnormalities are also found in aMCI patients, such as the raised local intrinsic functional connectivity between entorhinal cortex and MTL subregions [18]. Taken together, for AD and aMCI patients, these results indicated structural atrophy and abnormal functional activity in the MTL. However, most studies focused on the intrinsic activity of local brain regions. Given the complex pathologically mechanisms and probably alterations of whole-brain functional connectivity in AD, investigating how the functional connectivity changes at whole-brain wide levels in aMCI is significant.

Functional connectome studies equipped with graph theoretical approach model the human brain as a complex network with nodes as brain regions and edges as synchrony between the active regions, and investigate their topological architectures from a systematic perspective [19]. Shifted small-worldness network organization, changed connections between functional system and deficit in nodal centrality, have been suggested in functional networks in AD and aMCI [20, 21]. However, most brain network studies constructed the network models based on prior structural or functional parcellation, which might ignore the possible functional inhomogeneity within large brain regions. In contrast, high-resolution brain networks defining nodes at a voxel-level may unveil more detailed connectivity information especially for regions that contain multiple subdivisions [22, 23]. A recent study investigated the characteristics of voxel-wise whole-brain functional connectivity strength (FCS) in AD, which used a metric calculated as the sum of the connections for each voxel to capture its global functional integrity. They showed that the disrupted functional connectivity was mainly involving the densely connected hub regions in AD patients [24], which are also the brain regions selectively attacked by amyloid-β (Aβ) [25]. However, whether alterations of global functional integrity also emerge in patients with aMCI and whether these changes could have the potentially discriminating ability remains largely unknown.

In present study, we collected the resting-state fMRI and structural data from thirty-six aMCI patients and thirty-five age, gender and years of education-matched healthy controls (HCs). The atrophy degree of the MTL was estimated by medial temporal atrophy (MTA) visual rating scale and the whole brain functional network at the voxel level for each participant was constructed to investigate the abnormal global FCS in aMCI. We sought to find out: i) whether the patients with aMCI exhibit noteworthy atrophy and abnormal functional integrity in medial temporal cortices, and ii) if so, whether these functional abnormalities have the potential to distinguish patients with aMCI from HCs.

## Materials and methods

### Participants

Thirty-six aMCI patients were recruited from the Memory Clinic of the Department of Neurology, XuanWu Hospital of Capital Medical University, Beijing, China and thirty-five demographically matched HCs were recruited from communities by advertisements during June 2013 to March 2015. Patients with aMCI were diagnosed based on the criteria proposed by Petersen et al. and that has been described in our previous studies [3, 26, 27], which were: (1) memory loss complaint, preferably confirmed by an informant; (2) objective cognitive impairment in single or multiple domains, adjusted for age and education; (3) preservation of independence in functional abilities; (4) failure to meet the criteria for dementia according to the DSM-5 (Diagnostic and Statistical Manual of Mental Disorders, 5th edition); (5) the Clinical Dementia Rating (CDR) score is 0.5. Participants were excluded if met the following characteristics: (1) a history of stroke; (2) major depression (Hamilton Depression Rating Scale score > 24 points); (3) other central nervous system diseases that may cause cognitive impairment, such as brain tumors, Parkinson's disease, encephalitis and epilepsy; (4) cognitive impairment caused by traumatic brain injury; (5) systemic diseases, such as thyroid dysfunction, severe anemia, syphilis and HIV; (6) a history of psychosis or congenital mental growth retardation. HCs were recruited from communities through advertisements. The inclusion criteria were: (1) no complaint of memory loss and related disorders causing cognitive impairment; (2) CDR score is 0; (3) no severe visual or auditory impairment. All the participants were Han nationality and right-handed and carried on regular neuropsychological assessment, including mini-mental state examination (MMSE), Montreal cognitive assessment (MoCA), Rey Auditory Verbal Learning Test (AVLT), Clock Drawing Test (CDT) and MTA visual rating scale [8, 28]. We’d like to confirm that the clinical investigation involving human subjects are conducted in accordance with the ethical standards of the Helsinki Declaration. This study was also approved by the Research Ethics Review Board of XuanWu Hospital in the Capital Medical University (No. [2014]011). All of participants provided their written informed consent to participate in this study.

### MRI data acquisition

All participants were scanned using the 3.0 T Siemens scanner (Siemens, Erlangen, Germany) at XuanWu Hospital, Capital Medical University. Resting state functional images were collected using single shot echo-planar imaging (SS-EPI) sequence with the following parameters: repetition time (TR) / echo time (TE) = 2000/40 ms; flip angle = 90°; number of slices = 28; slice thickness = 4 mm; gap = 0.25 mm; voxel size = 4 × 4 × 4 mm^3^; and matrix = 64 × 64. Each scan lasted for 478s. Participants were asked to lie quietly in the scanner with their eyes closed without falling asleep and head motion. For registration purpose, high-resolution anatomical images were collected using a 3D magnetization-prepared rapid gradient echo (MPRAGE) T1-weighted sequence with the follow parameters: TR = 1900 ms, TE = 2.2 ms, inversion time (TI) = 900 ms, FA = 9°, number of slices = 176, slice thickness = 1 mm, voxel size = 1 × 1 × 1 mm^3^ and matrix = 256 × 256.

### Data analysis

#### Image preprocessing

Image preprocessing was performed using SPM8 (http://www.fil.ion.ucl.ac.uk/spm) and DPARSF (http://www.restfmri.net/forum/DPARSF) software [29]. The preprocessing procedures included discarding first 10 volumes, slice timing correction and head motion correction. All data used in this study satisfied the criteria of spatial movement in any direction < 3 mm or degree. To spatially normalize the fMRI data, for each individual, the T1-weighted image was first co-registered to the mean head-motion corrected fMRI image, segmented using DARTEL and transformed into Montreal Neurological Institute (MNI) space with the custom template generated from the group data. Notably, such a custom template could reduce the inaccuracy of spatial normalization due to the grey matter atrophy of aMCI patients and elderly HCs. The parameters estimated in DARTEL segmentation was applied to fMRI data and thus normalized fMRI data into MNI space. The functional data were further resampled to 3 mm isotropic voxels and spatially smoothed with a 4-mm full width half maximum (FWHM) Gaussian kernel. Then, the linear detrend and band-pass filtering (0.01–0.1Hz) was performed to reduce the effects of low-frequency drift and high-frequency noise. Finally, several nuisance signals including head motion (Friston’s 24 parameter model), global mean, and signals from the cerebrospinal fluid and white matter were regressed from the data.

#### Whole-brain functional connectivity strength analysis

Whole-brain functional connectivity strength was analyzed according the following procedure. Firstly, Pearson’s correlations between the time series of all pairs of voxels were computed to construct a whole-brain connectivity matrix for each participant. This computation was constrained within a gray matter (GM) mask, which was generated by setting a threshold of 0.2 on the mean map of all GM maps. Then we transformed individual correlation matrices to a z-score matrix using a Fisher r-to-z transformation to improve normality, and computed FCS as the sum of the connections (z-values) for each voxel. Given the ambiguous interpretation of negative correlations with removal of the global signal, we selected a threshold of *r* = 0.2 to conservatively restrict our analysis to positive correlations. Such a threshold was chosen to eliminate the voxels with weak correlations causing signal noise. Such a FCS metric is also considered to be the “degree centrality” of weighted networks in terms of graph theory [30, 31].

#### Discriminant analysis

To determine whether the FCS metric could serve as a potential biomarker for distinguishing individuals with aMCI from HCs, we performed a discriminant analysis based on the receiver operating characteristics (ROC) curve as follows. For each cluster showing significant between-group difference, we first extracted the mean FCS value of each subject and sorted these values. Then, a threshold was applied to classify the subjects into two groups according to their FCS values and the labels of each group (aMCI or HCs) were determined according to the direction of the between-group difference. The thresholds were selected from the smallest to the largest FCS values, and the classification accuracy, sensitivity and specificity were calculated under each threshold. Finally, a ROC was obtained based on the values of sensitivity and specificity for each cluster. Furthermore, the area under ROC curve (AUC) is used to quantitatively assess the diagnostic power of the FCS values, and the confidence interval of AUC to clarify their significance.

#### Statistical analysis

For the statistical analysis of the demographic information, two-sample *t* tests were performed to compare group differences in age, years of education, each neuropsychological test and MTA summed score, and Chi-squared test were used to compare group differences in gender. Group differences in FCS value were analyzed by using general linear model at the voxel level within the GM mask, of which the FCS was the dependent variable and group was the independent variable with age, gender and years of education as the covariates. The correction for multiple comparisons was performed using AlphaSim utility in AFNI based on Monte Carlo simulations (AFNI version: AFNI_16.2.05, Jul 22 2016). A threshold of *P* < 0.05 at voxel with cluster > 309 voxels was used to determine the significance level, corresponding to the corrected *P* < 0.05. The smooth kernel used in AlphaSim was 13.1548, 12.7669, 12.4638 mm FWHM, which was estimated using AFNI 3dFWHMx on the residual maps generated from the general linear model. Finally, general linear model was used to evaluate the relationships between FCS values of each brain region showing group differences and the clinical variables, with age, gender and years of education as the covariates in the aMCI group. The significant level for the correlation analysis was corrected using Bonferroni corrections for the six neuropsychological tests (*P* < 0.05/6).

#### Validation analysis

Considering that several choices of analysis strategies (e.g., connectivity threshold, head motion, and removal of global signal) may influence our main findings, we conducted the following procedures and re-compare the FCS between the two MCI groups: i) correlation thresholds. We used a single correlation coefficient threshold of 0.2 to eliminate weak correlations possibly arising from noise signal during the FCS analysis. To determine whether the results depended on the choices of correlation thresholds, we re-computed the FCS maps using other two different correlation thresholds (i.e., 0.1, and 0.3) and then performed statistical analysis, respectively. ii) Head motion. Several recent studies reported influences of head motion on RSFC [32–34]. Although no significant differences were found in the maximum movements at each direction between any pairs of the groups, we cautiously evaluated the effects of head motion on our results by calculating the frame-wise displacement (FD) of our data [34], and further compared the group difference with the FD served as an additional covariate. iii) Global signal regression (GSR). There is currently no consensus over whether the whole brain signal should be removed in preprocessing the R-fMRI data. Some studies suggested that the global signal was confounded with physiological noise [35] and should be removed [36], whereas several other studies [37, 38] indicated the GSR could introduce negative RSFC and thus alter the intrinsic correlation structure of the brain. To examine whether the process of GSR affected our results, the data was re-analyzed without GSR.

## Results

### Demographic and neuropsychological assessment of all the participants

The detailed demographic and neuropsychological information was presented in **Table 1**. There were no significant differences in age, gender, or years of education between aMCI patients and HCs group. However, compared with the HCs, the aMCI patients had significantly lower scores of the MMSE, MoCA, AVLT-immediate recall (AVLT-I), AVLT-delayed recall (AVLT-D) and AVLT-recognition (AVLT-R) (*P* < 0.001) and higher degree of MTA subjectively assessed (*P* < 0.05).

**Table 1:** Demographic and clinical assessment of all the participants.

	aMCI (n = 36)	HCs (n = 35)	*T* or *X* ^*2*^ Value	*P* Value
Age (years)	51–79 (67.3±7.6)	51–78 (64.8±6.2)	*T*_(69)_ = 1.538	0.129 ^a^
Gender (M/F)	17/19	13/22	*X*^*2*^_(1)_ = 0.739	0.390^b^
Education (years)	0–17 (9.8±3.8)	0–18 (11.1±4.4)	*T*_(69)_ = 1.250	0.215^a^
MMSE	17–30 (24.4±3.3)	22–30 (28.1±1.9)	*T*_(69)_ = 5.814	<0.001^a^
MoCA^c^	13–26 (19.7±3.9)	19–30 (26.8±2.3)	*T*_(69)_ = 9.637	<0.001^a^
CDT^d^	1–3 (2.2±0.7)	1–3 (2.7±0.5)	*T*_(67)_ = 3.803	<0.001^a^
AVLT-I	3.3–8.7 (5.8±1.3)	6–13.7 (9.4±1.7)	*T*_(69)_ = 9.637	<0.001^a^
AVLT-D	0–12 (3.3±2.7)	6–15 (10.3±2.5)	*T*_(69)_ = 11.465	<0.001^a^
AVLT-R	0–14 (7.0±3.7)	7–15 (11.9±2.4)	*T*_(69)_ = 6.703	<0.001^a^
MTA summed score	0–3 (1.3±0.7)	0–2 (0.9±0.5)	*T*_(69)_ *= 2*.*909*	<0.05 ^a^

Data are presented as the range of minimum–maximum (mean ± SD). aMCI, amnestic mild cognitive impairment; HCs, healthy controls; MMSE, Mini-Mental State Examination; MoCA, Montreal Cognitive Assessment; CDT, Clock Drawing Test; AVLT-I, auditory verbal learning test-immediate recall; AVLT-D, auditory verbal learning test-delayed recall; AVLT-R, auditory verbal learning test-recognition; MTA, medial temporal atrophy.

^a^The *p* value was obtained by two-sample two-tailed *T* test.

^b^The *p* value was obtained by two-tailed *Pearson* chi-square test.

^c^MoCA included 36 aMCI and 33 HCs.

^d^CDT included 34 aMCI and 35 HCs.

### Disrupted FCS in patients with aMCI

**Fig 1:**
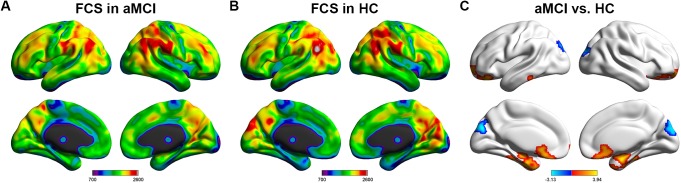
The FCS in aMCI and HCs and the group differences. (A) and (B) These two pictures showed the mean FCS both in aMCI and HCs. The color bar represents the FCS value for each group and the warm color means higher FCS value. (C) This picture indicated the group differences between aMCI patients and HCs on FCS value. The color bar at the bottom represents *T* value (*p* < 0.05, Cluster > 309 voxels). FCS, functional connectivity strength; aMCI, amnestic mild cognitive impairments; HC, healthy controls.

As illustrated in **Fig 1A and 1B**, regions with higher FCS value were mostly located in the association cortices, including the posterior cingulate cortex/precuneus (PCC/PCU), dorsolateral prefrontal cortex (DLPFC), lateral parietal lobe, and visual cortex in both groups, which is consistent with previous findings. Comparing with HCs, the aMCI group showed significantly increased FCS predominantly in the bilateral hippocampus (HIP) and parahippocampus gyri (PHG) extending to orbitofrontal cortices and significantly lower FCS in the bilateral superior occipital gyrus (SOG) and cuneus (CUN) (**Fig 1C** and **Table 2**).

**Table 2:** Group differences of FCS value between aMCI and HCs.

No.	Brain regions	Brodmann area	Cluster size	Peak MNI coordinate			Max *T* score
				x	y	z	
1	R.HIP/FG/PHG/TPOmid;	11/25/38/47	914	27	-6	-24	3.940
	B.ORBsup/REC/ORBmid						
2	L.FG/ITG/TPOmid/HIP/PHG	20/36/38	337	-30	-9	-33	3.413
3	B.CUN/SOG	19/18	334	-6	-81	27	-3.132

Significance level: *P* < 0.05, cluster > 309 voxels, AlphaSim corrected *P* < 0.05. B, bilateral; L, left; R, right; HIP, Hippocampus; FG, fusiform gyrus; PHG, parahippocampal gyrus; TPOmid, middle Temporopolar; ORBsup, superior orbitofrontal cortex; REC, rectal gyrus; ORBmid, middle orbitofrontal cortex; ITG, inferior temporal gyrus; CUN, cuneus; SOG, superior occipital gyrus.

### FCS-based classification analysis

**Fig 2:**
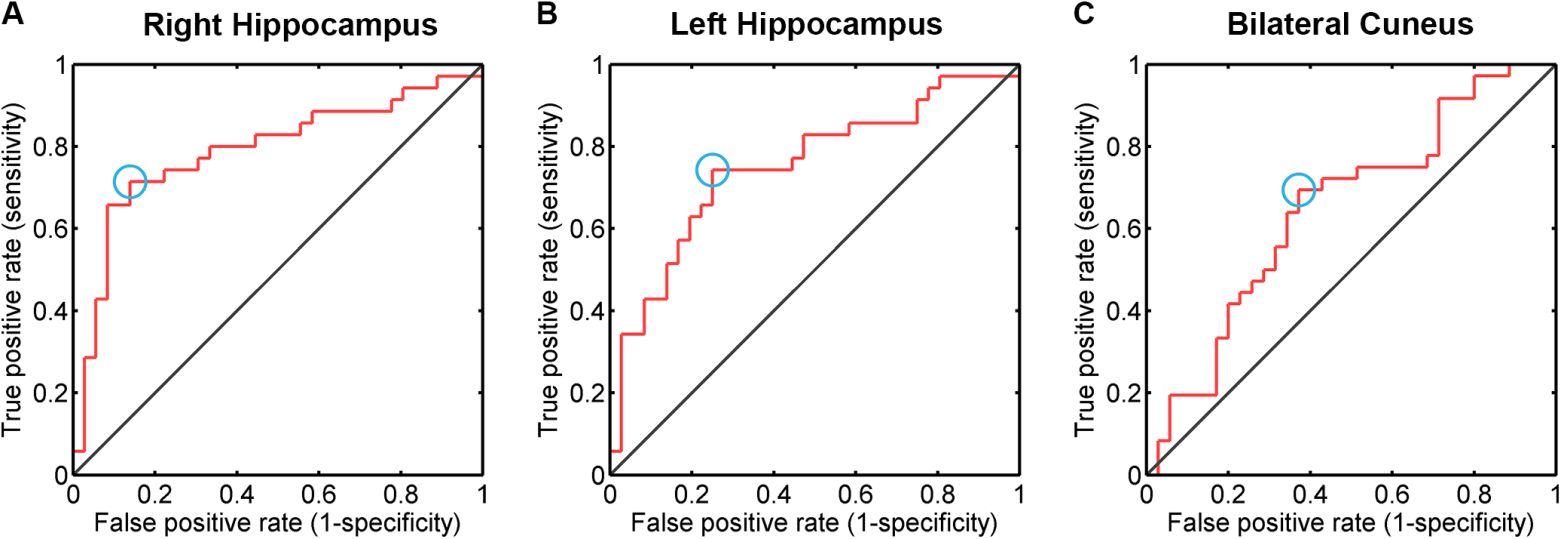
FCS-based classification analysis. The ROC of FCS in the right hippocampus (A), the left hippocampus (B), and the bilateral cuneus (C) in distinguishing individuals with aMCI from HCs. FCS, functional connectivity strength.

Using the ROC analysis approach, we estimated the discriminative power of the mean FCS within the left and right hippocampus, as well as the bilateral cuneus (**Fig 2**). As comparing to the random situation (AUC = 0.5), all these regions exhibited a significantly higher power in distinguishing individuals with aMCI from HCs (all *P* < 0.031). In detail, the right hippocampus had the highest classification power, with the AUC of the ROC of 0.790 (confidence interval: 0.678, 0.901) and those for left hippocampus and bilateral cuneus were 0.752 (confidence interval: 0.636, 0.867), and 0.649 (confidence interval: 0.520, 0.778), respectively.

### Correlation between FCS and neuropsychological characteristics in aMCI

**Fig 3:**
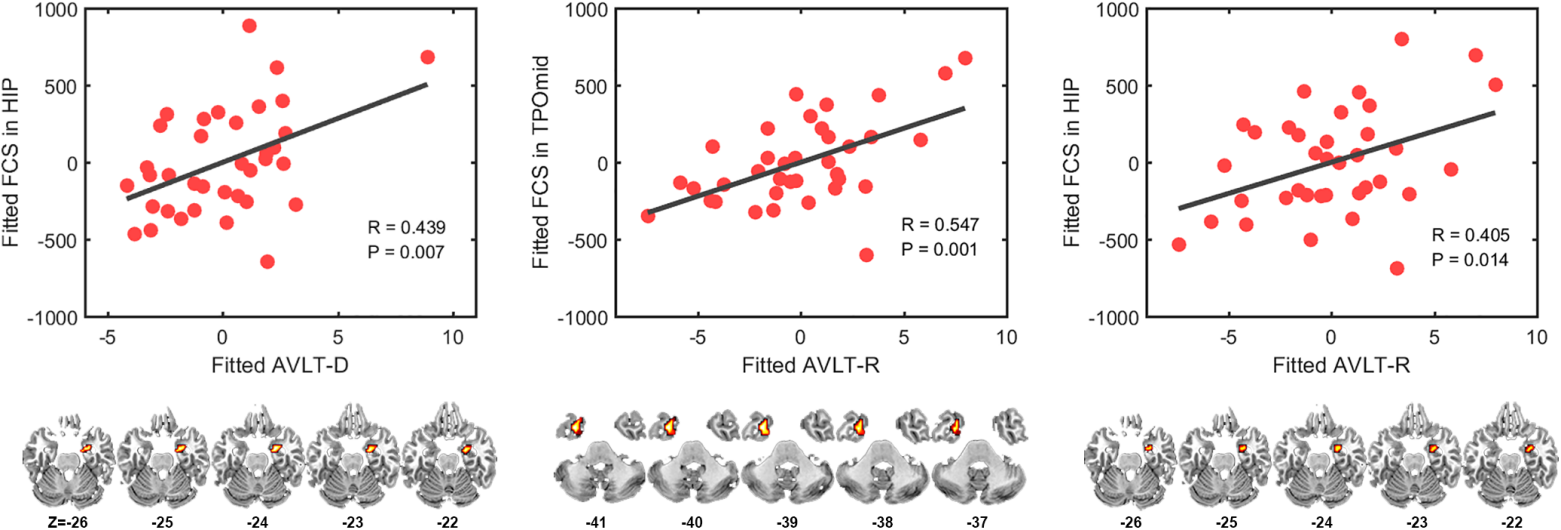
Correlation between FCS and clinical scores in aMCI patients. (A) This picture showed marginally positive correlation between FCS value of right hippocampus and AVLT-D (R = 0. 439, P = 0.007, corrected-P = 0.042); (B) This picture showed the positive correlation between FCS value of left TPOmid and AVLT-R (R = 0.547, P = 0.001, corrected-P = 0.006); (C) This picture showed the marginally positive correlation between FCS value of right hippocampus and AVLT-R (R = 0.405, P = 0.014, corrected-P = 0.084). FCS, functional connectivity strength; aMCI, amnestic mild cognitive impairments; HIP, hippocampus; TPOmid, temporal pole (middle), AVLT-D, auditory verbal learning test-delayed recall; AVLT-R, auditory verbal learning test-recognition.

We found significantly positive correlation in aMCI between the FCS in right hippocampus and AVLT-D (R = 0. 439, corrected-P = 0.042) and between the left temporal pole (middle) and AVLT-R (R = 0.547, corrected-P = 0.006). The FCS in the right hippocampus was marginally correlated with the AVLT-R (R = 0.405, corrected-P = 0.084) (**Fig 3**).

### Validation

**Fig 4:**
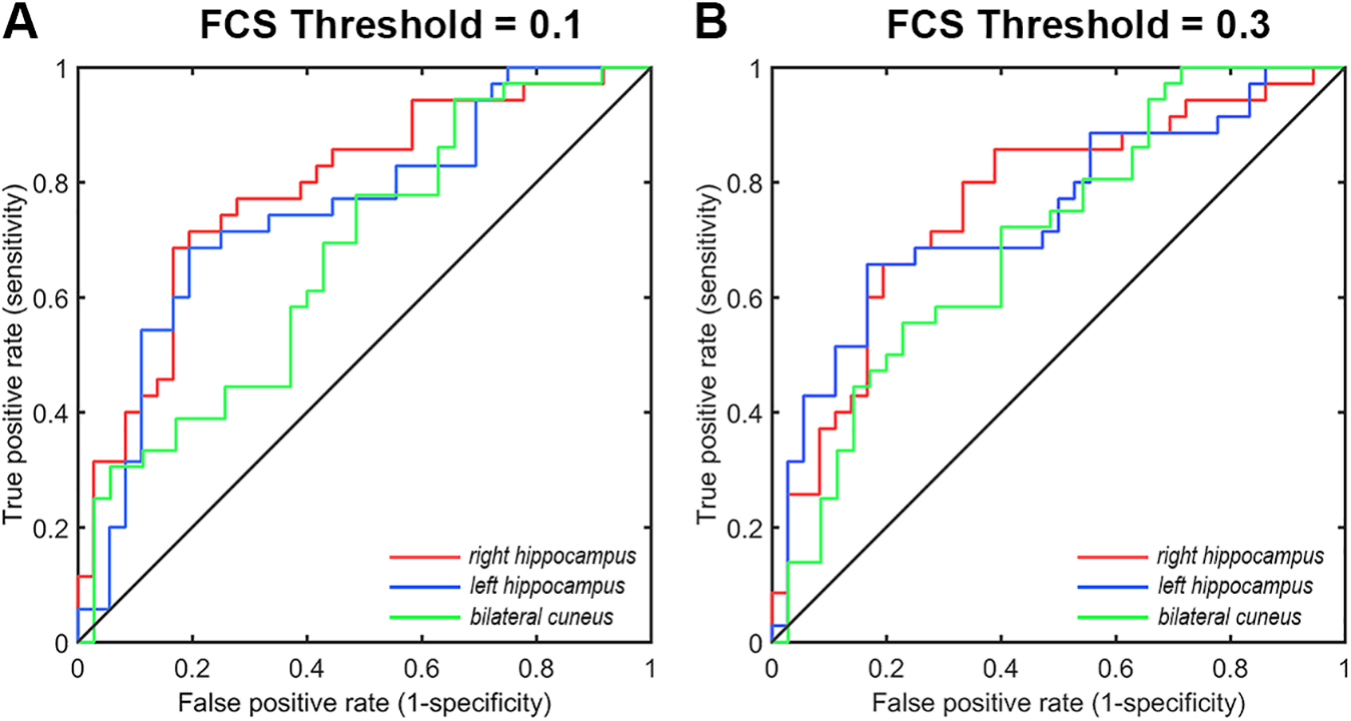
Validation of FCS-based classification analysis for different FCS thresholds. The ROC of FCS in the right hippocampus (red), the left hippocampus (blue), and the bilateral cuneus (green) in distinguishing individuals with aMCI from HCs under FCS threshold of 0.1 (A) and 0.3 (B). FCS, functional connectivity strength.

We evaluated the reproducibility of our main findings under several different image preprocessing procedures and data analysis strategies. We found that connectivity thresholds did not have effect on our main result, all of the between-group differences remained under the thresholds of 0.1 and 0.3. Notably, the left and right hippocampus, as well as the bilateral cuneus also exhibited significant higher power in distinguishing individuals with aMCI from HCs under these two thresholds (all *P* < 0.013). Under threshold of 0.1, the right hippocampus had the highest classification power, with the AUC of the ROC of 0.787 (confidence interval: 0.680, 0.894) and those for left hippocampus and bilateral cuneus were 0.748 (confidence interval: 0.632, 0.864), and 0.671 (confidence interval: 0.545, 0.796), respectively. Under threshold of 0.3, AUC of ROC in the right hippocampus, left hippocampus and bilateral cuneus were 0.763 (confidence interval: 0.651, 0.876), 0.746 (confidence interval: 0.630, 0.862), and 0.702 (confidence interval: 0.581, 0.824), respectively (**Fig 4**). The two group had no difference on frame-wise head motion (p = 0.396), and the results remained unchanged after adding frame-wise head motion as an additional covariate. Additionally, the between-group differences could not be identified in case the global signal was not regressed during the preprocessing, implying the effects of different imaging processing strategies on detecting pathological differences.

## Discussion

In this study, we investigated the FCS characteristics in patients with aMCI and found that: (i) compared with HCs, aMCI patients had significantly higher degree of MTA scores and increased FCS value predominantly in bilateral MTL regions and orbitofrontal cortices, while decreased FCS mainly in bilateral occipital cortices; (ii) the right hippocampus exhibited a highly discriminative power in distinguishing aMCI from HCs; (iii) the FCS values of right hippocampus and left temporal pole were positively correlated with the AVLT scores. Together, these results demonstrated the changed whole-brain connectivity integrity from a network perspective and suggested the potentially distinguishing power for identifying aMCI from HCs.

### Spatial distribution of FCS in aMCI and HCs

The measurement of FCS captures the global functional integration information of the network node and brain regions with higher FCS value were usually considered as hub regions. Previous studies have demonstrated that the hub regions of the functional brain network were mostly located in the DMN regions (e.g., PCC, DLPFC, angular gyrus and inferior parietal lobe) and visual cortex (e.g., CUN and SOG) [20, 25, 39–41]. Most of these regions are also identified in the present study. Researchers suggested that hub regions are responsible for integrating information among multiple distributed regions when high-level cognitive tasks are executed [42], which is of great values for achieving communication with great efficiency and reducing the cost of energy especially through the long-distance information transmission [43]. Our results demonstrated a similar spatial distribution of FCS map in the two groups, suggesting that even under the condition of disease, hub regions may still maintain central placement and preserve its functional integration roles in brain network. This was confirmed by previous studies from Wang et al. and Li et al., which investigated the similarity in spatial distribution of hub regions between aMCI and HCs [20, 44]. Moreover, Dai et al. further found that even in the AD group, the distribution of hub regions is nearly consistent with HCs [24], indicating again a relative preservation of crucial roles played by those regions. It is noteworthy that recent findings suggest that both the structural and functional hubs were keen to be attacked in neuropsychiatric diseases [45]. More importantly, the hub regions are selectively attacked in AD and aMCI patients and are associated with the accumulation of high amounts of Aβ deposits [46], suggesting the importance of brain hubs in the AD pathology.

### Simultaneity of functional disruption and compensation in aMCI

Further, comparing to HCs, aMCI had significantly increased FCS value predominantly in bilateral MTLs and orbitofrontal cortices, while decreased FCS mainly in bilateral occipital cortices, including CUN and SOG. MTL, including hippocampus, parahippocampal gyrus, fusiform gyrus, entorhinal cortex, plays a critical role in the cognitive process, especially in the storage and retrieval of the episodic memory [5]. Researchers have confirmed that AD pathology deposits initially in the MTL and progresses to posterior parts of the brain and then spreads to the anterior parts, such as medial prefrontal cortex [6]. Therefore, disruption of MTL is tightly related with the decline of episodic memory. In prior studies, the relationship between abnormalities of functional activity in MTL and memory has been investigated [47] and some of them have revealed that MCI patients can exhibit increased fMRI responses in MTL when executing memory-related tasks [11, 12, 14], indicating a compensatory response to accumulating AD pathology. Similarly, using rs-fMRI, some studies also show increased activity or connectivity in components of MTL, which is also related to the cognitive function [16, 18, 48, 49], such as raised intrinsic activity in MTL subregions associated at-trend with impaired delayed recall [17] and the positive correlations between basal functional connectivity within the ATN and performance on declarative memory tasks in patients [50]. However, there are some discrepancies among different studies [26, 51], possibly due to the heterogeneity of aMCI [52] and different types of tasks performed [11].

The possible mechanism of aberrant MTL activity in aMCI patients may be as follows: Firstly, “hippocampus disconnection hypothesis”, which emphasizes that reduced main cortical input to hippocampus may lead to disinhibition-like changes of intrahippocampal activity, causing the elevated synchrony of hippocampus circuit activity [17, 18, 53]. Pasquini et al. also found that increased local intrinsic functional connectivity in the hippocampus was associated with decreased global functional connectivity within the DMN [16]. Secondly, the increased functional connectivity has been proposed to be a compensatory reallocation or recruitment of additional neural resources for against the accumulation of AD pathology for MCI patients [50]. In our study, we found the significantly higher MTA scores in aMCI patients compared to the HCs group, providing evidence that increased functional connectivity in MTL compensated the structural disruption. Another evidence is the higher baseline cerebral blood flow of hippocampus in the prodromal AD [54]. In addition, it is possible that increased FCS in MTL may be a performance of cognitive reserve or abnormal plasticity of neuron [12].

Meanwhile, we also found SOG and CUN regions with decreased FCS value. They are the main parts of the primary visual cortex, which receive information from the homolateral geniculate nucleus and transmits information through two primary pathways, the ventral stream and the dorsal stream. These two pathways are associated with complex cognitive function, such as memory, motion, representation of object locations, etc [55]. Although the loss of epidemic memory is the most typical syndrome of aMCI, deficits in performance of other cognitive domains may still exist simultaneously [4], such as visual memory decline, abnormal visuospatial perception, and illusion etc. MCI patients had poorer performance in the long-term visual memory comparing to HCs, especially those who had converted to AD dementia [56]. These deficits could be attributed to the distribution of neurodegeneration in AD involving the visual pathways, such as atrophy in the left middle occipital gyrus [57]. Pathologically, neurofibrillary tangles and neuritic senile plaques increasing steadily were identified through primary to associative visual cortices [58], which may be the possible reason of abnormal functional connectivity between visual cortex and other regions. In addition, SOG and CUN are also important components of the association cortex and they are also preferentially attacked in aMCI patients.

In our study, FCS value at the right hippocampus has the potential power of identifying aMCI patients from HCs with a relatively high accuracy. This is consistent with the observation published recently that neuroanatomical asymmetries in hippocampus could predict the progression from aMCI to dementia [59], and such FCS abnormalities may serve as a potential neuroimaging marker to monitor the disease progression as well.

### Limitations and future directions

There are several limitations of this study. First, we found that the FCS in the hippocampus and cuneus offered discrimination power in distinguishing aMCI from HCs, which could be substantially changed in independent samples. Future studies including independent datasets for multi centers might provide valuable evidence for estimating the reliability of the current findings. Second, the present study was a cross-sectional design and longitudinal studies from HCs to different stages of AD, including subjective cognitive decline (SCD) [60, 61], aMCI and AD dementia, should be conducted to reflect changing patterns of the whole-brain functional connectivity. Third, many studies have confirmed the disruption of structural brain network in AD/aMCI patients [62, 63]. Thus, multi-modality MRI techniques combining functional and structural MRI, as well as diffusion MRI data are of great importance to comprehensively reveal the structural and functional abnormalities in prodromal AD. Finally, recent studies have revealed genetic effects on brain mechanism in AD pathology, such as APOE ε4 allele [64]. Future imaging studies involving genetic information might provide a novel perspective in providing potential neuroimaging biomarkers for early diagnosis of AD.

## Conclusion

The present study demonstrated that both prominent atrophy and increased functional activity in MTL were shown in aMCI patients, suggesting a pathological mechanism of simultaneous disruption and compensation during the stage at high risk for AD. Furthermore, such neuroimaging abnormalities may have the potential discrimination for identifying prodromal AD. Together, the present study provided further evidence of functional connectome alterations in aMCI, which would deepen our understanding for the brain mechanism of AD pathology at the high-risk stage.

## Supporting information

S1 Table Demographic and neuropsychological data of aMCI patients. (DOCX)

S2 Table Demographic and neuropsychological data of healthy controls. (DOCX)

S3 Table Degree of MTA in all the participants. (DOCX)

S1 File The mean FCS map across aMCI patients. (NII)

S2 File The mean FCS map across healthy controls. (NII)

S3 File T map derived from two sample t-test between aMCI and HC groups. (NII)

S4 File Clusters with significant between-group differences. (NII)

S5 File Multiple comparison threshold determined by AlphaSim. (1D)

S1 Excel Data used for correlation analysis, including the clinical variables and extracted FCS values. (XLSX)
