# Supplementary material for: Abnormal global functional network connectivity and its relationship to medial temporal atrophy in patients with amnestic mild cognitive impairment
Source: PLoS One. 2017 Jun 26;12(6):e0179823. doi: 10.1371/journal.pone.0179823 (PMC5484500; doi:10.1371/journal.pone.0179823)
Supplement: S1 Table — (DOCX) [file pone.0179823.s001.docx]

**S1 Table. Demographic and neuropsychological data of aMCI patients.**

| Subject Number | Age (years) | Gender (M/F) | Education (years) | CDR | CDT | MMSE | MoCA | AVLT-I | AVLT-D | AVLT-R |
| --- | --- | --- | --- | --- | --- | --- | --- | --- | --- | --- |
| 1* | 72 | F | 6 | 0.5 | / | 25 | 18 | 4.33 | 3 | 7 |
| 2 | 54 | F | 15 | 0.5 | 3 | 24 | 20 | 4 | 0 | 1 |
| 3 | 66 | M | 9 | 0.5 | 3 | 25 | 22 | 6 | 2 | 6 |
| 4 | 66 | F | 9 | 0.5 | 1 | 23 | 16 | 5.33 | 0 | 0 |
| 5 | 75 | F | 15 | 0.5 | 2 | 24 | 13 | 4.33 | 3 | 6 |
| 6 | 53 | F | 6 | 0.5 | 2 | 17 | 13 | 4.67 | 0 | 4 |
| 7 | 67 | M | 15 | 0.5 | 2 | 28 | 25 | 8 | 5 | 12 |
| 8 | 77 | F | 9 | 0.5 | 3 | 26 | 19 | 5.33 | 6 | 9 |
| 9 | 76 | F | 0 | 0.5 | 2 | 24 | 15 | 5 | 0 | 1 |
| 10 | 63 | M | 11 | 0.5 | 2 | 27 | 24 | 5 | 4 | 10 |
| 11 | 72 | F | 15 | 0.5 | 2 | 23 | 21 | 5.67 | 5 | 6 |
| 12 | 76 | M | 8 | 0.5 | 2 | 29 | 22 | 7.33 | 6 | 10 |
| 13 | 72 | M | 17 | 0.5 | 3 | 26 | 24 | 5 | 5 | 10 |
| 14 | 71 | M | 7 | 0.5 | 2 | 23 | 18 | 4 | 0 | 6 |
| 15 | 57 | F | 8 | 0.5 | 2 | 18 | 15 | 4.33 | 0 | 2 |
| 16 | 62 | M | 6 | 0.5 | 2 | 25 | 25 | 4.67 | 3 | 9 |
| 17 | 58 | M | 11 | 0.5 | 2 | 20 | 14 | 4 | 3 | 7 |
| 18 | 51 | M | 9 | 0.5 | 1 | 23 | 16 | 7 | 1 | 4 |
| 19 | 66 | F | 9 | 0.5 | 3 | 24 | 24 | 7.33 | 5 | 9 |
| 20 | 74 | M | 13 | 0.5 | 2 | 26 | 23 | 7.67 | 7 | 8 |
| 21 | 59 | F | 7 | 0.5 | 1 | 21 | 17 | 6.67 | 5 | 7 |
| 22 | 73 | M | 9 | 0.5 | 3 | 26 | 21 | 6.33 | 5 | 10 |
| 23* | 67 | M | 9 | 0.5 | / | 30 | 22 | 5.33 | 6 | 8 |
| 24 | 71 | F | 8 | 0.5 | 3 | 25 | 16 | 7.33 | 5 | 5 |
| 25 | 74 | F | 6 | 0.5 | 2 | 22 | 20 | 6.67 | 4 | 8 |
| 26 | 66 | F | 6 | 0.5 | 3 | 21 | 17 | 7.67 | 5 | 6 |
| 27 | 71 | M | 15 | 0.5 | 3 | 27 | 24 | 5.33 | 3 | 7 |
| 28 | 69 | F | 12 | 0.5 | 3 | 29 | 24 | 7.33 | 4 | 14 |
| 29 | 62 | M | 9 | 0.5 | 2 | 28 | 24 | 5.67 | 5 | 14 |
| 30 | 70 | M | 9 | 0.5 | 1 | 30 | 19 | 5.67 | 1 | 7 |
| 31 | 57 | F | 12 | 0.5 | 1 | 25 | 23 | 8.67 | 12 | 13 |
| 32 | 79 | F | 17 | 0.5 | 2 | 20 | 18 | 5 | 0 | 2 |
| 33 | 58 | F | 12 | 0.5 | 3 | 27 | 26 | 7 | 0 | 5 |
| 34 | 75 | F | 4 | 0.5 | 3 | 18 | 14 | 6.67 | 2 | 6 |
| 35 | 78 | M | 12 | 0.5 | 1 | 26 | 15 | 3.33 | 0 | 1 |
| 36 | 66 | M | 9 | 0.5 | 2 | 24 | 23 | 6.33 | 2 | 12 |

aMCI, amnestic mild cognitive impairment; CDR, Clinical Dementia Rating; CDT, Clock Drawing Test; MMSE, Mini-Mental State Examination; MoCA, Montreal Cognitive Assessment; AVLT-I, auditory verbal learning test-immediate recall; AVLT-D, auditory verbal learning test-delayed recall; AVLT-R, auditory verbal learning test-recognition

*Suject No.1 and subject No.23 lack the score of CDT.
