# Supplementary material for: Abnormal global functional network connectivity and its relationship to medial temporal atrophy in patients with amnestic mild cognitive impairment
Source: PLoS One. 2017 Jun 26;12(6):e0179823. doi: 10.1371/journal.pone.0179823 (PMC5484500; doi:10.1371/journal.pone.0179823)
Supplement: S2 Table — (DOCX) [file pone.0179823.s002.docx]

**S2 Table. Demographic and neuropsychological data of healthy controls.**

| Subject Number | Age (years) | Gender (M/F) | Education (years) | CDR | CDT | MMSE | MoCA | AVLT-I | AVLT-D | AVLT-R |
| --- | --- | --- | --- | --- | --- | --- | --- | --- | --- | --- |
| 1 | 57 | M | 12 | 0 | 3 | 27 | 27 | 10.67 | 11 | 14 |
| 2 | 69 | M | 6 | 0 | 3 | 29 | 27 | 9.67 | 12 | 14 |
| 3 | 60 | F | 6 | 0 | 3 | 27 | 28 | 6 | 6 | 10 |
| 4 | 70 | F | 9 | 0 | 1 | 27 | 26 | 8.67 | 10 | 12 |
| 5 | 58 | F | 9 | 0 | 3 | 27 | 26 | 6.67 | 9 | 10 |
| 6 | 59 | F | 6 | 0 | 3 | 29 | 26 | 7 | 8 | 10 |
| 7 | 61 | M | 11 | 0 | 3 | 27 | 25 | 9 | 9 | 13 |
| 8 | 70 | F | 8 | 0 | 3 | 30 | 27 | 7.67 | 9 | 15 |
| 9 | 61 | F | 10 | 0 | 3 | 30 | 30 | 11.67 | 11 | 13 |
| 10 | 69 | F | 12 | 0 | 2 | 30 | 25 | 9.67 | 12 | 11 |
| 11 | 61 | F | 12 | 0 | 3 | 30 | 29 | 8.33 | 9 | 12 |
| 12 | 67 | M | 15 | 0 | 2 | 29 | 25 | 10.33 | 15 | 15 |
| 13 | 67 | F | 15 | 0 | 3 | 27 | 26 | 9.33 | 6 | 12 |
| 14 | 62 | F | 9 | 0 | 3 | 29 | 25 | 8.67 | 10 | 13 |
| 15 | 78 | M | 6 | 0 | 3 | 27 | 29 | 10 | 11 | 11 |
| 16 | 71 | M | 12 | 0 | 3 | 26 | 29 | 8.67 | 8 | 7 |
| 17 | 67 | F | 17 | 0 | 2 | 29 | 27 | 12 | 15 | 15 |
| 18 | 70 | F | 16 | 0 | 3 | 29 | 29 | 13.67 | 15 | 15 |
| 19 | 73 | F | 15 | 0 | 3 | 26 | 25 | 9.67 | 12 | 14 |
| 20 | 59 | F | 9 | 0 | 2 | 29 | 27 | 8.33 | 10 | 11 |
| 21 | 74 | F | 0 | 0 | 3 | 22 | 19 | 9.67 | 10 | 9 |
| 22 | 60 | M | 9 | 0 | 3 | 28 | 28 | 8 | 7 | 10 |
| 23 | 61 | F | 9 | 0 | 3 | 30 | 30 | 8.33 | 11 | 14 |
| 24 | 64 | F | 1 | 0 | 3 | 24 | 22 | 9 | 7 | 8 |
| 25* | 58 | M | 13 | 0 | 3 | 30 | / | 11.33 | 13 | 14 |
| 26* | 73 | M | 18 | 0 | 3 | 30 | / | 11.33 | 13 | 12 |
| 27 | 72 | F | 17 | 0 | 3 | 30 | 30 | 10 | 12 | 14 |
| 28 | 51 | F | 14 | 0 | 3 | 29 | 27 | 9 | 9 | 11 |
| 29 | 68 | M | 13 | 0 | 2 | 30 | 29 | 6 | 8 | 8 |
| 30 | 61 | F | 15 | 0 | 3 | 27 | 28 | 11.67 | 14 | 14 |
| 31 | 60 | F | 9 | 0 | 3 | 28 | 27 | 10.33 | 8 | 7 |
| 32 | 61 | F | 8 | 0 | 2 | 30 | 26 | 9 | 11 | 12 |
| 33 | 58 | M | 18 | 0 | 3 | 30 | 26 | 11.33 | 11 | 15 |
| 34 | 66 | M | 13 | 0 | 3 | 27 | 28 | 9.33 | 9 | 13 |
| 35 | 71 | M | 15 | 0 | 2 | 26 | 25 | 7.67 | 8 | 10 |

HCs, healthy controls; CDR, Clinical Dementia Rating; CDT, Clock Drawing Test; MMSE, Mini-Mental State Examination; MoCA, Montreal Cognitive Assessment; AVLT-I, auditory verbal learning test-immediate recall; AVLT-D, auditory verbal learning test-delayed recall; AVLT-R, auditory verbal learning test-recognition

*Suject No.25 and subject No.26 lack the score of MoCA.
