# Supplementary material for: Abnormal global functional network connectivity and its relationship to medial temporal atrophy in patients with amnestic mild cognitive impairment
Source: PLoS One. 2017 Jun 26;12(6):e0179823. doi: 10.1371/journal.pone.0179823 (PMC5484500; doi:10.1371/journal.pone.0179823)
Supplement: S3 Table — (DOCX) [file pone.0179823.s003.docx]

**S3 Table. Degree of MTA in all the participants**

| aMCI (n = 36) | | | HCs (n = 35) | | |
| --- | --- | --- | --- | --- | --- |
| Left (score) | Right (score) | Summed score | Left (score) | Right (score) | Summed score |
| 2 | 1 | 1.5 | 1 | 0 | 0.5 |
| 1 | 1 | 1 | 2 | 1 | 1.5 |
| 2 | 2 | 2 | 1 | 0 | 0.5 |
| 1 | 1 | 1 | 1 | 0 | 0.5 |
| 3 | 1 | 2 | 1 | 1 | 1 |
| 1 | 1 | 1 | 0 | 0 | 0 |
| 2 | 1 | 1.5 | 1 | 0 | 0.5 |
| 1 | 2 | 1.5 | 1 | 1 | 1 |
| 2 | 2 | 2 | 1 | 0 | 0.5 |
| 1 | 2 | 1.5 | 0 | 1 | 0.5 |
| 2 | 1 | 1.5 | 1 | 1 | 1 |
| 1 | 2 | 1.5 | 1 | 1 | 1 |
| 1 | 2 | 1.5 | 0 | 1 | 0.5 |
| 2 | 1 | 1.5 | 1 | 1 | 1 |
| 0 | 0 | 0 | 2 | 1 | 1.5 |
| 1 | 1 | 1 | 2 | 2 | 2 |
| 1 | 1 | 1 | 1 | 1 | 1 |
| 1 | 1 | 1 | 2 | 1 | 1.5 |
| 0 | 1 | 0.5 | 1 | 1 | 1 |
| 3 | 3 | 3 | 1 | 1 | 1 |
| 0 | 1 | 0.5 | 0 | 1 | 0.5 |
| 1 | 1 | 1 | 1 | 0 | 0.5 |
| 1 | 1 | 1 | 0 | 0 | 0 |
| 0 | 1 | 0.5 | 0 | 0 | 0 |
| 2 | 1 | 1.5 | 0 | 1 | 0.5 |
| 2 | 1 | 1.5 | 1 | 1 | 1 |
| 1 | 2 | 1.5 | 1 | 1 | 1 |
| 0 | 1 | 0.5 | 1 | 1 | 1 |
| 1 | 0 | 0.5 | 0 | 1 | 0.5 |
| 1 | 1 | 1 | 1 | 1 | 1 |
| 0 | 0 | 0 | 1 | 1 | 1 |
| 2 | 2 | 2 | 1 | 2 | 1.5 |
| 1 | 1 | 1 | 1 | 1 | 1 |
| 1 | 1 | 1 | 1 | 2 | 1.5 |
| 3 | 3 | 3 | 2 | 1 | 1.5 |
| 2 | 2 | 2 |  |  |  |

MTA, medial temporal atrophy; aMCI, amnestic mild cognitive impairment; HCs, healthy controls; Summed score is the mean value on both sides.
